# Serum Calcium Level at Diagnosis Can Predict Lethal Prostate Cancer Relapse

**DOI:** 10.3390/jcm13164845

**Published:** 2024-08-16

**Authors:** Zsolt Fekete, Patricia Ignat, Henrietta Jakab, Nicolae Todor, István Péter László, Alina-Simona Muntean, Sebastian Curcean, Adina Nemeș, Dumitrița Nuțu, Gabriel Kacsó

**Affiliations:** 1 Department of Oncology, “Iuliu Hatieganu” University of Medicine and Pharmacy, 400015 Cluj-Napoca, Romania; suteu.patricia@umfcluj.ro (P.I.); ja_henriett@yahoo.com (H.J.); sebastian.curcean@gmail.com (S.C.); adina.nemes@umfcluj.ro (A.N.); nutudumitrita@yahoo.com (D.N.); gabi.kacso@gmail.com (G.K.); 2 Oncology Institute, 400015 Cluj-Napoca, Romania; todor@iocn.ro (N.T.); istvanlaszlo@yahoo.com (I.P.L.); muntean.alina@yahoo.fr (A.-S.M.); 3 Amethyst Radiotherapy Center, 407280 Cluj-Napoca, Romania

**Keywords:** serum calcium, prostate cancer, follow-up, tumor markers, early intervention, prognostic factors, predictive factors, metastases

## Abstract

**Background/Objectives**: The most important prognostic factors in curatively treated prostate cancer are T and N stage, histology, grade group and initial PSA. A recent study found that men with blood calcium levels at the high end of the normal range are over two-and-a-half times more likely to develop fatal prostate cancer than those with lower calcium levels. However, there is limited evidence regarding the prognostic value of calcium levels at the time of prostate cancer diagnosis. We aimed to determine whether a calcium level in the upper range of normal values has any prognostic value in curatively treated prostate cancer. **Methods**: We conducted a retrospective analysis of 84 consecutive patients with prostate cancer who underwent curative-intent radiotherapy—either as primary treatment or adjuvant therapy—using external beam radiotherapy with or without brachytherapy. We analyzed all pertinent prognostic factors that could potentially impact disease-free survival. **Results**: The study revealed that calcium levels at diagnosis significantly predict disease-free survival, whereas the initial PSA level did not hold prognostic significance—likely due to interference from benign prostatic hyperplasia. **Conclusions**: If our findings are validated, calcium levels at the time of prostate cancer diagnosis could be incorporated into future predictive and prognostic models.

## 1. Introduction

According to the WHO, prostate cancer (PC) is the third most common cancer world-wide, accounting for approximately 10% of all cancer cases, and it is the second leading cause of cancer-related deaths [1]. In men over the age of 65 years, the histological incidence rate is nearly 60% [2]. Differences in incidence rates are almost 160-fold between the populations at the highest rate (France, Guadeloupe, 157.5) and the populations with the lowest rate (Bhutan, 1.1), reflecting risk factors and screening practices [3]. Prostate cancer, similar to breast cancer, is an elusive neoplasm regarding its etiology. If in other tumor types, risk factors such as smoking, infections (HPV, HBV, HCV, EBV, Helicobacter pylori, etc.) and pollutants could easily explain most of the cases, in prostate cancer, diet-related conditions, race and ethnicity, and a Western lifestyle are the only major risk factors [4]. Newer, hypothetical etiologic factors are anaerobic bacteria, found through genetic studies and identification from urine and semen cultures [5].

Prostate cancer is one of the most curable cancers, if diagnosed early. Still, a certain percentage of cases will relapse despite the most meticulous staging and prompt treatment. It is still difficult to triage patients in the more and less aggressive groups. The D’Amico and derived systems (NCCN, NICE, GUROC, EAU, and AUA) are the most widely used systems to assess prostate cancer progression risk. These categorize patients into three recurrence risk groups based on factors such as initial prostate specific antigen (iPSA) levels, Gleason grades and tumor stages, as originally described by D’Amico et al. [6]. Including additional variables in the risk stratification tools often enhances granularity, but it also introduces greater complexity and can hinder usability. This trade-off is evident in more recent risk scores and nomograms [7].

One of the most extensive analysis and validation study of different prognostic tools has been performed by Zelic et al. [8].

There are newer, readily available prognostic classifiers, such as multiparametric prostate magnetic resonance imaging (mpMRI) with fusion-targeted biopsy, cribriform growth in Gleason score 7 prostate cancer, the “third type” of tumor grade in the biopsy (added as the third denominator to the Gleason score), neutrophil-to-lymphocyte ratio and PSA density. DNA tests promise the ultimate prognostication [9].

Is further study of prognostic tools a necessity? We believe that the answer is yes, until virtually all low-risk-labeled prostate cancers can be cured. But what if new classifiers show a higher risk of relapse and/or death? Here is our second task to fulfill: readily address micrometastases with new, efficient treatment modalities.

Our study, building upon a significant prior publication [10], aimed to assess the prognostic significance of pretreatment calcium (Ca). Notably, our approach differs from that of Skinner et al., who analyzed prediagnostic Ca levels approximately 10 years before the diagnosis of fatal prostate cancer. In our investigation, we studied Ca levels at the time of prostate cancer diagnosis.

Total calcium levels are usually measured in serum or plasma. Serum levels are normally between 8.8 and 10.2–10.4 mg/dL (2.2 to 2.6 mmol/L) in healthy subjects [11].

The role of calcium in prostate cancer cell homeostasis has been summarized by Ardura et al. [12].

## 2. Materials and Methods

Our study, conducted at a tertiary academic cancer center (Oncology Institute, Cluj, Romania), analyzed consecutive patients diagnosed with prostate cancer between June 2005 and March 2011. These patients received curative treatment involving radiotherapy (primary or adjuvant), which included external beam, brachytherapy, or both, along with neoadjuvant and adjuvant hormone therapy.

The inclusion criteria were as follows:

- Primary confirmed prostate adenocarcinoma;
- Clinical or pathological stage I, II or III (T1–4, N0) [13];
- Curative-intent treatment;
- At least 10 years of follow-up;
- Proper TNM staging and proper follow-up procedures.

The exclusion criteria were as follows:

- Multiple synchronous cancers;
- Clinical stage IV;
- Diseases which might interfere with Ca homeostasis.

The study database was constructed by reviewing 207 files related to patients who underwent curative treatment for prostate cancer at our institution. After applying the inclusion criteria, a total of 84 patients were selected over a nearly 6-year period. The exclusion criteria led to the removal of 121 patients due to insufficient follow-up (less than 10 years), and an additional two patients were excluded due to the presence of oligometastatic disease.

Within the pretreatment window of one month, we gathered available calcium (Ca) values. Additionally, we recorded alkaline phosphatase (ALP) values as a surrogate marker for bone metastasis, whenever they were available. Notably, our institutional protocol did not mandate routine measurement of either Ca levels or ALP. Serum Ca was assessed using a colorimetric assay in our laboratory, while ALP measurements were conducted via spectrophotometry.

All patients underwent proper staging with either MRI of the pelvis or endorectal ultrasound (or both) and CT scan of the pelvis, abdomen, and thorax. If the iPSA level exceeded 20 ng/mL, or if the patient had a Gleason score of 8, 9 or 10, or if they presented with bone pain, they also underwent a bone scan. Additionally, bone MRI or plain radiographs were obtained when necessary. The staging procedures respected the international guidelines for middle resource centers. Choline- or PSMA-PET was not available for patients treated in this period in Romania.

Restaging for the purpose of our study was performed according to the AJCC Cancer Staging Manual, Eighth Edition, from 2017 [13].

All data were collected in a FileMaker 10 database and analysis was performed through Excel Microsoft Office 2007.

The overall survival (OS) and disease-free survival (DFS) were determined through the Kaplan–Meier method. Survival differences were evaluated through the log-rank test. To analyze the influence of initial Ca on DFS, we chose a cut-off value with the aid of an ROC curve and a chi-squared test (using Yate’s correction). The cut-off value was chosen by the minimum distance of the ROC curve to point (0, 1). The odds ratio was calculated with the chi square test.

The confidence intervals were estimated at the 95% confidence level. The statistical significance was defined by a value of *p* ≤ 0.05.

## 3. Results

The age of the patients ranged from 48 to 77 years, with a median of 67 years. The median follow-up was 14.1 years, or 169.3 months (range 10.3–22.4 years). All patients (pts) had the most common type of prostate adenocarcinoma, the acinar subtype. All patients were cN0 or pN0. The patient characteristics are shown in Table 1.

Most of our patients (seventy-five subjects,, i.e., 89.3%) were treated with primary radiotherapy (RT), and only nine patients (10.7%) underwent combined-modality treatment, consisting of surgery (eight prostatectomy, one cystoprostatectomy) and adjuvant or salvage radiotherapy.

Six of our patients underwent surgical castration (7.1%). A total of additional 63 patients (75%) underwent hormone therapy with complete androgen blockade (LHRH, i.e., luteinizing hormone releasing hormone, agonist-antagonist plus bicalutamide, 14 patients) or androgen deprivation therapy with LHRH agonist–antagonist only (49 patients). Since most of our patients were high risk, the length of the hormone blockade was intended to last 2–3 years, unless contraindication or side-effects; at the end of analysis, we observed that only a total of 22 (26.2%) patients received hormone therapy for more than 1 year, in addition to the 6 patients who underwent surgical castration. The median duration of hormone therapy was 6 months, with a minimum of 2 months and a maximum of 36 months. Hormone therapy was started 2–3 months before the radical treatment to aid local procedures. Although 80 of our patients had high-risk disease, only 28 of these (35%) were prescribed or could maintain long-term hormone therapy (11 patients for 24 months and 7 patients for 36 months).

For patients receiving external beam radiotherapy (EBRT) as the only local treatment procedure (52 patients, comprising 61.9% of the total group), the radiation dose ranged from 70 Gy to 78 Gy, with 2 Gy/day, 5 days a week. The vast majority of EBRT-only subjects, 43/52, i.e., 82.7%, received a dose of either 74 Gy/37 fractions (16 patients) or 76 Gy/38 fractions (27 patients), under actual guidelines at the time of treatment, with respecting doses for organs at risk (OARs). Adjuvant or salvage EBRT after prostatectomy was administered in a dose of 50 Gy/25 fractions in 1 patient, 66 Gy/33 fractions in 3 pts, 70 Gy/35 fraction in 3 pts and 76 Gy/38 fractions in 2 pts.

Almost one-third of our patients (23 subjects, 27.4%) received combined EBRT-BT (brachytherapy) treatment to boost the primary tumor and decrease local toxicity. The BT consisted of a permanent I^125^ implant of 110 Gy, followed at 1–2 months by EBRT to cover possible or proven extraprostatic extension, with or without lymph node areas, when necessary, based on the Partin tables [14]. BT was performed by an expert radiation oncologist (G.K.) with extensive previous experience in Nice, France. Only EBRT was reimbursed by state insurance, not BT.

For the EBRT-only patients, we employed a 3D technique (dual energy Varian and Siemens Primus LINACs of 6 and 15–16 MV) without fiducial markers, the only type of treatment available at that time at our institution (before the introduction of intensity modulated radiotherapy—IMRT—and stereotactic radiotherapy). Treatment was applied with a bladder-filling protocol and an empty rectum. For patients with BT implants, we could identify the “seeds” on portal imaging to guide positioning.

There is a lack of data regarding prostate volume for patients who did not receive brachytherapy (BT) as part of their treatment. Consequently, we do not have information on PSA density for the entire patient group. Additionally, there is inadequate documentation of perineural and lymphatic invasion in the biopsy reports.

During the follow-up period, 32 out of 84 patients (38.1%) experienced a relapse of prostate cancer. Among these patients, most (26 out of 32, or 81.3%) presented bone metastases as part of their relapse. In two cases, there were additional metastases to the peritoneum or lymph nodes, along with parenchymatous organs (such as the liver and lung). Two patients had biochemical-only failure, characterized by a PSA rise of 2 ng/mL or more above the post-EBRT nadir. One patient had lymphatic-only relapse in a right obturatory lymph node, which was successfully treated with surgery. Additionally, three patients had local relapse only.

The relapse of prostate cancer occurred at a median of 7.8 years (range 0.3–11.9).

The two patients presenting biochemical-only failure passed away due to second cancers (metastatic colon cancer and advanced urothelial bladder cancer) at 6 years from the diagnosis of prostate cancer. These were the only deaths from a second malignancy in our group, although two more patients presented a second malignancy, i.e., transverse-colon adenocarcinoma and germ-cell testicular cancer, which were cured.

At the conclusion of the 14-year follow-up period, more than half of the patients (44 out of 84, or 52.4%) had passed away. However, only 12 of these deaths (14.3%) were attributed to prostate cancer. The majority of deaths were related to cardiovascular and infectious diseases (30 out of 84, or 35.7%). Among these, two patients with stable relapsed disease at the time of death succumbed to causes other than prostate cancer. Ten patients remained alive at the end of the follow-up period, despite having relapsed prostate cancer. Unfortunately, this retrospective study could not definitively determine the impact of hormone therapy on cardiovascular deaths.

In the context of RT ± surgery, the most significant long-term side effect was urethral stricture, affecting 15 out of 84 patients (17.9%). Additionally, three patients experienced urinary incontinence, and two patients had G3 rectal bleeding, which was managed through LASER coagulation.

**Figure 1:**
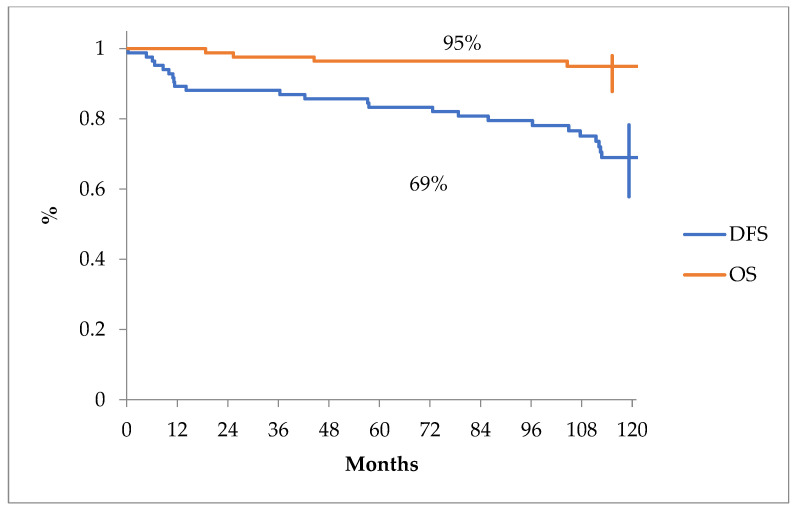
Overall survival and disease-free survival projected at 10 years for all patients from our analysis.

The overall survival and disease-free survival curves can be seen in Figure 1.

The primary tumor stage did not predict outcomes. Clinical stages—considering a combination of TNM stage, Gleason score and PSA levels—showed differences in disease-free survival (DFS). Specifically, stage I and II patients exhibited better survival rates compared to stage III patients: 81% versus 64%, respectively, but the *p*-value was 0.36, indicating no significant difference (NS).

There was also a tendency for higher disease-free survival for patients with a Gleason score of less or equal to 6 (prognostic group 1) versus those with a score of more than 6 (prognostic groups 2 through 5), 76% versus 64%, *p* = 0.42, NS.

**Figure 2:**
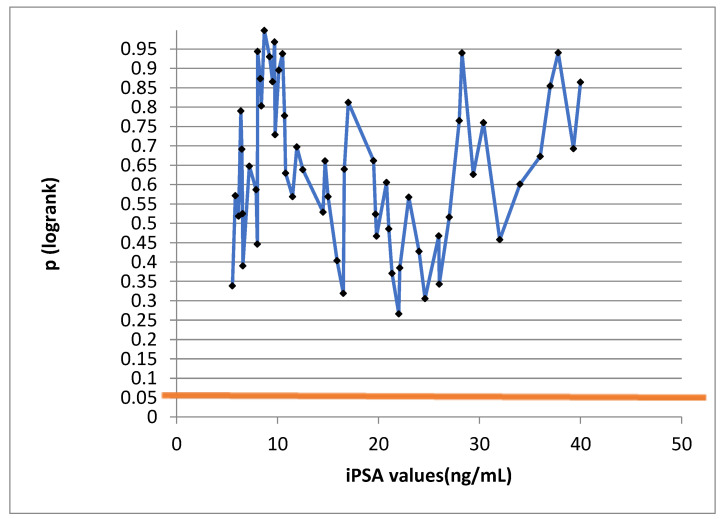
Log-rank test to correlate disease-free survival and PSA; *p*-values do not fall below 0.05.

The iPSA values were not of prognostic significance, as seen on the log-rank test in Figure 2. The broken line unites individual PSA values. No value reached statistical significance, i.e., it was not below the value of 0.05, read on the *Y*-axis.

The age of patients at diagnosis did not have prognostic value in our case series.

After searching for classical prognostic factors, we looked at serum calcium and alkaline phosphatase at diagnosis. Calcium levels were only available for 57 patients (Table 2), while alkaline phosphatase levels were available for 69 patients.

**Figure 3:**
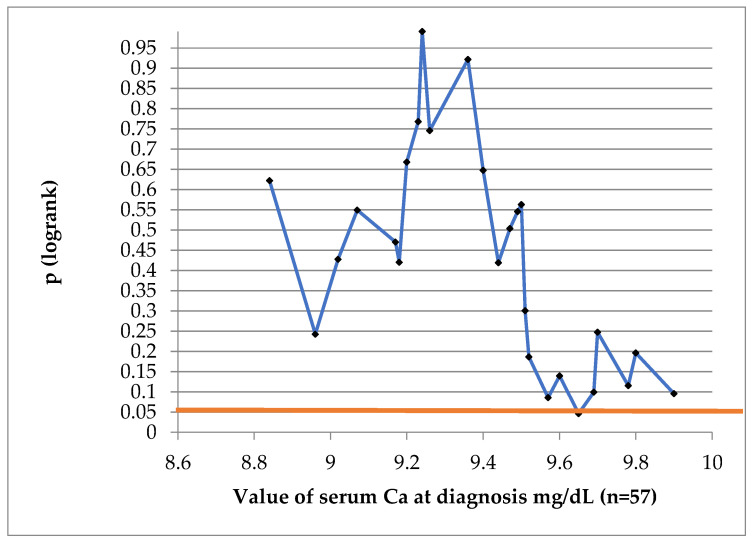
Log-rank test to establish if Ca at diagnosis is a prognostic factor for DFS. (Blue line represents the *p* value at different Ca cut-off values; red straight line represents conventional statistical significance, of 0.05).

To find a value of Ca which might be prognostic for DFS at 10 years, we have performed a log-rank test, indicated in Figure 3. The value of 9.65 mg/dL was of prognostic significance.

**Figure 4:**
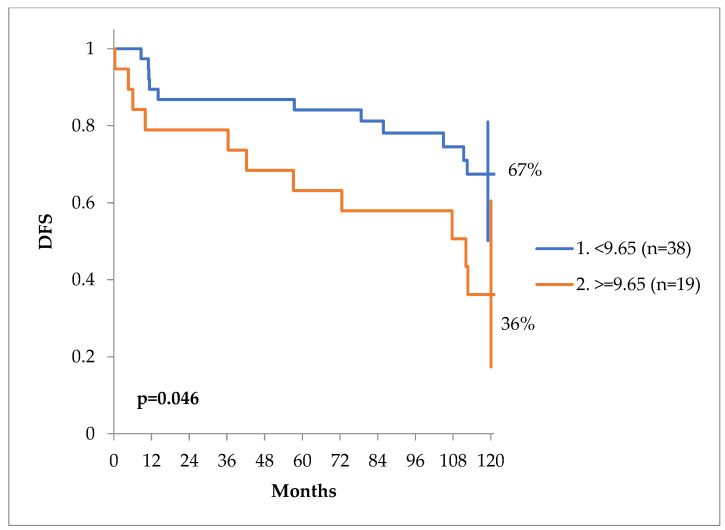
DFS at 10 years of patients with a serum calcium level of more or less than 9.65 mg/dL.

The DFS curves can be seen in Figure 3. At 10 years, the DFS of patients with calcium values less than 9.65 mg/dL was 67% versus only 36% for patients with a value of equal or more than 9.65 mg/dL, *p* = 0.046 (Figure 4).

In Table 3, the calcium values for patients with relapse and those without relapse are compared. While there was no statistical correlation between calcium values and the crude rate of relapse, a significant difference was observed at the level of disease-free survival (DFS), as shown above.

Alkaline phosphatase levels were available for 69 patients. At our laboratory, normal values were considered to be between 44 and 129 international units IU/L. The distribution of alkaline phosphatase values can be observed in Table 4.

**Figure 5:**
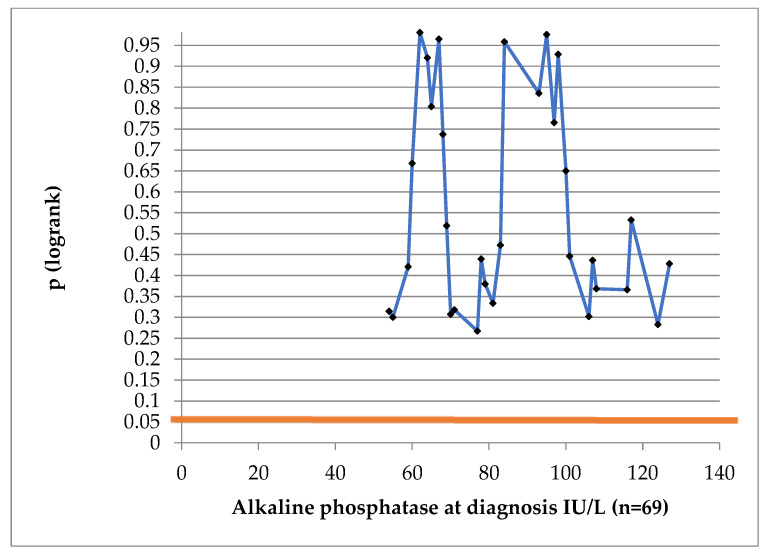
Log-rank test to determine the prognostic value of alkaline phosphatase. (Blue line represents the *p* value at different AP cut-off values; red straight line represents conventional statistical significance, of 0.05.)

At no cut-off value was ALP statistically significant for DFS (log-rank test in Figure 5).

## 4. Discussion

The disease-free survival was significantly influenced by the value of the serum calcium at diagnosis, at a cut-off value of 9.65 mg/dL. Several studies have demonstrated the pre-diagnosis value of calcium in predicting death from prostate cancer, but no research group, as of our knowledge, has proven any relationship of serum calcium at diagnosis with relapse of prostate cancer.

In the publication of Skinner et al. [10], the study team examined the association be-tween serum Ca levels and prostate cancer death using a prospective cohort, the National Health and Nutrition Examination Survey (NHANES). Comparing men in the top tertile with men in the bottom tertile of serum Ca, the multivariable-adjusted relative hazard ratio (HR) for fatal prostate cancer was 2.68 (95% confidence interval, 1.02–6.99; *p* = 0.04). Of note, Ca in the upper tertile, and not hypercalcemia, was the factor determining an increased risk of death, since only very few patients had hypercalcemia, similar to our study (only three patients in our patient group). Serum calcium was determined in the Skinner cohort at an average of 9.9 years before the primary diagnosis of prostate cancer. Their finding of a >2.5-fold increase in the risk of death from prostate cancer for men in the highest tertile of serum calcium was comparable in magnitude with the increased risk associated with family history (2.5×).

Skinner et al. confirmed their results on another cohort as well [15].

The concentration of serum ionized Ca (the biologically active fraction total Ca, representing about 50% of the total amount) is tightly regulated by parathyroid hormone (PTH) and usually does not deviate by more than 2% from its stabilized value. In our study, the values of total Ca have not been adjusted to the albumin level, since all patients had normal levels of serum albumin, and in non-malnourished patients, total serum calcium has a good correlation to PTH [16].

Recently, Kim et al. [17] have proven that in prostate cancer, the levels of PTH are higher than the levels in matched controls. The average PTH level was 41.67 pg/mL in PC patients, compared to only 27.06 pg/mL in the matched benign hyperplasia (BPH) group, *p* < 0.001, irrespective of PSA levels (≤20 or >20 ng/mL), Gleason score (≤7 or ≥8) or stage (≤T3 or ≥T4). The average postoperative PTH level (26.93 pg/mL) was significantly lower than the pre-operative PTH level (36.71 pg/mL) in the same patients who underwent radical prostatectomy.

A PTH increase and thus a parallel Ca rise is in some way connected to or induced by prostate cancer, without the presence of any clinical bone metastases.

In relation to other malignancies without bone metastases, Datta et al. [18] demonstrated that elevated serum calcium levels are associated with more advanced melanoma stages. Specifically, the odds ratio (OR) for higher stages increased by 60% for every 1.0 mg/dL rise in albumin-corrected calcium.

PTH or a similar hormone might be induced by prostate and other types of tumor cells.

The increase in PTH levels may not always indicate a true increase; in other words, it can be a false positive result. In some cases, parathyroid hormone-related protein (PTHrP), a protein with N-terminal similarity to PTH, can falsify the PTH results [19]. Physiologically, PTHRP produced by osteoblasts is a regulator of bone formation [20]. When measuring PTH, not only PTHrP but also PTH-like peptides can potentially affect the accuracy of determination [21,22]. Thus, “high PTH” might reflect high PTHRP or high PTH-like peptides. Indeed, in a diverse population of cancer patients with hypercalcemia due to malignancy, plasma PTHrP levels were elevated in 76% of subjects [23]. PTHrP is expressed by prostate cancer, and it increases cancer cell growth and enhances the osteolytic effects of prostate cancer cells. PTHrP gene expression is upregulated via a transcriptional mechanism by epidermal growth factor (EGF), which is secreted by prostate cancer cells [24].

Peterson et al. [25]. had a similar approach as ours, but their study had a negative result: pretreatment Ca value did not predict biochemical relapse. Their study differed from ours, since (1) their patient cohort consisted of only salvage radiotherapy patients, where the Ca levels might have been influenced by previous radical prostatectomy and (2) the team did not study the DFS.

Skinner et al. also proposed that serum PTH stimulates prostate growth in men without clinical prostate cancer [26].

PTH and PTH-like proteins are seemingly both a predictive factor for prostate cancer and an aggressivity-inducing feature when prostate cancer is already present.

A large majority our patients (92.9%) had stage T3 disease, reflecting the lack of screening in Romania. Initial PSA was high as well (with a median value of 15.9 ng/mL, with almost 40% of patients having values higher than 20 ng/mL), but there were no clinical metastases and 60/84 (71.4%) of our patients did not relapse.

More than 95% of our patients have been high-risk patients based on classical prognostic factors. Our results regarding the value of Ca and the apparent uselessness of PSA as a prognostic factor must be interpreted in the setting of more advanced and high-risk prostate cancer but still N0. A multivariate analysis was not possible due to a limited number of subjects in each risk category. It is likely that elevated PSA levels in some patients were attributable to benign prostatic hyperplasia rather than advanced prostate cancer. A limitation of our study was the absence of measurement for the benign hyperplasia component of prostate volume, which would affect the PSA levels.

Another limitation of our study is the apparent underdiagnosis of Gleason 7 and higher prostate cancer, since there is a discrepancy between the high percentage of cT3 disease (92.9%) and the high percentage of Gleason 6 tumors (45.2%). More precisely, out of the 38 patients with low-grade (Gleason less or equal than 6) tumors, 34 were high risk based either on T3 stage or a PSA higher than 20 ng/mL, or both.

Burke [27] discusses the controversy surrounding the classification of Gleason score 6 prostate cancer. The debate centers on whether such low-grade cancers should be labeled as cancer due to their minimal risk of causing death. Some researchers propose renaming these low-risk lesions to avoid the psychological and treatment burdens associated with the cancer diagnosis and suggest the term “indolent lesions of epithelial origin (IDLE)” to reflect the low morbidity and mortality risk. However, others argue that despite the low risk, a Gleason score of 6 still exhibits malignant characteristics, such as tissue invasion, and should not be dismissed as non-cancer. Conversely, there is fear and overtreatment driven by the cancer label. The author concludes that while Gleason score 6 tumors are not typically lethal, they should still be recognized as cancer.

Yamazaki et al. [28]. define a high-risk low-grade [HRLG] group, consisting of patients with a Gleason score ≤ 6 and PSA over 20 ng/mL. Following BT with or without EBRT, the frequency of clinical failure in the HRLG group was 0% in their analysis, after a follow-up of 5 years. The authors suggest that these patients might be considered for less aggressive treatment due to their favorable outcomes.

In our study, out of the 38 patients with low-grade (Gleason less or equal to 6) tumors, 34 were high risk based either on T3 stage or PSA higher than 20 ng/mL, or both. The fact that 11 out of these 34 high-risk low-grade (HRLG) patients relapsed (32.4%) shows that there is an underestimation of the Gleason grade through biopsy in our cohort, since low-grade tumors should recur only extremely rarely, as seen above.

Alkaline phosphatase values lacked any prognostic significance in our analysis.

Li et al. published in 2018 the first meta-analysis regarding the level of ALP and DFS in PC [29]. The authors included 33 retrospective cohort studies, regarding both localized and metastatic PC. Elevated serum ALP influenced both DFS (HR = 1.60, 95% CI: 1.13–2.26) and OS. In the individual cohorts there was large variability in the upper normal limit of the ALP, or cut-off value for analysis, ranging from 90 to 620 U/L. In our laboratory, the upper normal limit was 129 IU/L and only nine patients (10.7%) exhibited elevated ALP levels. Consequently, the low cut-off value used by us and the limited number of subjects with this feature yielded no statistically significant impact of ALP levels on the disease-free survival (DFS) in this cohort.

Overall, the primary limitation of our study is the small sample size. However, despite this constraint, our findings on the role of serum calcium are statistically significant, indicating its potential as a robust prognostic factor.

We acknowledge the well-documented Gleason migration from the initial biopsy to the final specimen. There is an increase in the Gleason score from the initial biopsy to the prostatectomy in around half of the reported cases [30], although downgrading is also possible in around 10% of the patients [31]. In our study, we relied on the Gleason score obtained after surgery, which is considered more accurate for primary surgery patients. Notably, primary surgery patients constitute only 10.7% of our total patient cohort (9 out of 84). While this case-mix limitation exists, we believe it does not compromise the main conclusions of our study.

The quest to identify new circulating, spermatic and biopsy-identified biomarkers in prostate cancer is still open for early detection, prediction and management [32,33]. De-cipher^®^, Prolaris^®^ and Oncotype DX^®^ are genetic risk-stratifying tools which have already entered clinical practice and are recommended as optional [34]. The major drawbacks of currently available prognostic tools are biomarker diversity, limited number of patients enrolled in each study without further multicentric validation, and disregard of accepted clinical tools (the D’Amico-derived tools and nomograms) [35].

The future application of our study would be validation on a larger retrospective cohort from a different center and a prospective analysis, consisting of calcium measurement at diagnosis and at follow-up, along with PSA, PTH and other bone metabolism-related substances, such as osteprotegerin (OPG).

OPG is part of the tumor necrosis factor (TNF) receptor superfamily. It is a soluble osteoclastogenesis inhibitor; thus, its primary role is in bone remodeling. It binds to the receptor activator of nuclear factor kappa B ligand (RANKL). In cancer, OPG plays a significant role, affecting multiple hallmarks of cancer, tumor survival, epithelial-to-mesenchymal transition (EMT), neo-angiogenesis, invasion and metastasis [36]. In the study of Brown et al., serum OPG levels were significantly increased in advanced PC compared to non-metastatic PC and locally recurring PC. No significant correlation was found between serum OPG levels and PSA levels [37].

## 5. Conclusions

Total serum calcium level at the time of diagnosis, at a cut-off value of 9.65 mg/dL, in non-metastatic prostate cancer treated with curative-intent radiotherapy had a prognostic significance for disease-free survival. Our cohort consisted of mostly high-risk, high-PSA patients. Based on our understanding and search of the literature, we are the first to publish this concept along with a study showing positive associations. If the role of initial calcium is confirmed in future reports, it might be included in multiparametric prognostication tools.

## Figures and Tables

**Table 1:** Patient characteristics.

Variable	Number	(%)
T (tumor) stage		
T1	1	1.2
T2	5	6
T3	63	81
T4	15	17.8
Gleason group (score)		
Grade group 1: ≤ 6 (3 + 3)	38	45.2
Grade group 2: 7 (3 + 4)	23	27.4
Grade group 3: 7 (4 + 3)	10	11.9
Grade group 4: 8 (4 + 4), (3 + 5), (5 + 3)	9	10.7
Grade group 5: 9 or 10 (4 + 5), (5 + 4), (5 + 5)	3	3.6
No grading	1	1.2
iPSA		
<10 ng/mL	30	35.7
10–20 ng/mL	20	23.8
>20 ng/mL	33	39.3
None found	1	1.2
Stage		
I	1	1.2
II	22	26.2
III	61	72.6
Risk groups based on original D′Amico criteria		
Low risk	2	2.4
cT1–cT2a AND Grade Group 1 AND PSA <10 ng/mL		
Intermediate risk	2	2.4
cT2b–cT2c AND Grade Group 2 or 3 AND PSA 10–20 ng/mL		
High risk	80	95.2
cT3a OR Grade Group 4 or Grade Group 5 OR PSA >20 ng/mL		
Treatment modality		
EBRT only	52	61.9
BT followed by EBRT	23	27.4
Surgery with adjuvant EBRT	8	9.5
Surgery with salvage EBRT	1	1.2
Neoadjuvant/Adjuvant hormone therapy or surgical castration	69	82.1
Surgical castration	6	8.7
Complete androgen blockade	14	20.3
Androgen deprivation therapy with LHRH agonist-antagonist only	49	71

**Table 2:** Calcium values across the patient group.

Calcium Values	Number	Percentage
Lower than normal (<8.8 mg/dL)	9	15.8
Considered normal (8.8–10.2 mg/dL)	45	78.9
Higher than normal (>10.2 mg/dL)	3	5.3
Total	57	

**Table 3:** Calcium in relapsed and non-relapsed cases.

Patients	Average Ca Value (mg/dL)	Min–Max Value (mg/dL)
With relapse (biochemical, local, nodal and/or metastatic)	9.55	7.65–10.30
No relapse	9.28	7.45–14.80
Total	57	

**Table 4:** Alkaline phosphatase values across the patient group.

Alkaline Phosphatase Values	Number	Percentage
Lower than normal (<40 IU/L)	2	2.9
Considered normal (40–129 IU/L)	58	84.1
Higher than normal (>129 IU/L)	9	13
Total	69	

## Data Availability

The data presented in this study are available on request from the corresponding author. The data are not publicly available due to patient privacy.
